# Bilateral spontaneous internal carotid artery dissection; a treatment dilemma: A case report and literature review

**DOI:** 10.1016/j.ijscr.2024.110526

**Published:** 2024-10-28

**Authors:** Ahmed Almumtin, Fedah Farhan Almutairi, Amro Hajja, Nancy Mohamed Darwish, Samer Koussayer

**Affiliations:** a King Faisal Specialist Hospital and Research Center, Riyadh, Saudi Arabia; b College of Medicine, Alfaisal University, Riyadh, Saudi Arabia

**Keywords:** Bilateral carotid artery dissection, Conservative management, Antiplatelet therapy, Vascular surgery, Case report

## Abstract

**Introduction:**

Spontaneous carotid artery dissection in association with exercise is well known. A bilateral occurrence is a rarely reported finding. SVS guidelines discussed the role of antiplatelets and anticoagulants in management, however, management of dissections associated with pseudoaneurysm formation is still controversial. Herein, we report a case of walking-induced spontaneous bilateral carotid artery dissection complicated by a unilateral pseudoaneurysm formation treated conservatively with satisfactory outcome.

**Case presentation:**

A 41-year-old male presented with a sudden severe headache, blurred vision, and transient right upper limb weakness after a long walk. Initial CT angiography showed bilateral carotid artery dissection. The patient was managed conservatively with antiplatelet therapy and close follow up. Follow-up imaging showed gradual resolution of the dissection bilaterally and a stable right internal carotid artery pseudoaneurysm. Five years later, the patient remained asymptomatic with shrinking ICA pseudoaneurysm.

**Discussion:**

Bilateral spontaneous carotid artery dissection is a rare condition that can present with stroke-like symptoms, including visual changes and motor deficits, as observed in the presented case. Conservative management with antiplatelet therapy showed a favorable outcome, aligning with the recommendations of the SVS guideline. The role of antiplatelet therapy in managing pseudoaneurysms as a complication of dissection remains controversial, with endovascular interventions generally preferred despite reported complications. However, in our case, conservative management with antiplatelet therapy showed satisfactory outcomes, as supported by some recent studies.

**Conclusion:**

The reported case presents a rare occurrence of spontaneous bilateral carotid artery dissection complicated by a unilateral pseudoaneurysm formation. Conservative treatment with antiplatelets is associated with good outcomes, and potentially in highly selected patients complicated with pseudoaneurysm formation.

## Highlights

• Bilateral spontaneous carotid artery dissection often presents with atypical neurological symptoms.
• Prompt neurological assessment and radiographic investigations are crucial for accurate diagnosis.
• Conservative management with antiplatelet therapy proved to be effective.
• Conservative management of pseudoaneurysm is valid in highly selected patients.
• Regular follow-up with imaging is essential to monitor resolution.

## 1 Introduction

Carotid artery dissection occurs when a tear forms in the wall of the carotid arteries, allowing blood to enter and separate the layers [1]. The incidence of spontaneous carotid artery dissection is around 2.5–2.9 cases per 100,000 individuals [2]. The etiology is often unclear, but risk factors include cervical trauma, hypertension, connective tissue disorders, and recent infections [3].

Spontaneous dissections are typically unilateral, while spontaneous bilateral carotid artery dissection is rare [4]. Patients with carotid artery dissection often present with pain such as headache and neck pain, or ischemic symptoms such as transient ischemic attack (TIA) or stroke [4]. Diagnosis is made by imaging, with CT angiography, MR angiography, or albeit invasive, conventional angiography demonstrating a characteristic appearance of an intimal flap, intramural hematoma, or pseudoaneurysm [5].

Management remains controversial, with both antiplatelet and anticoagulation therapy used to prevent thromboembolic complications [5]. Left untreated, carotid artery dissection may lead to stenosis, vessel occlusion, aneurysm formation, stroke, or persistent TIAs [1]. In this case, we report a rare occurrence of bilateral spontaneous carotid artery dissection in a 41-year-old male managed conservatively with a positive outcome. This case report has been reported in line with the PROCESS guidelines and SCARE criteria [6,7].

## 2 Case presentation

A non-smoker, 41-year-old male was referred to our hospital from another institution, presenting to us five months after an episode of a sudden severe headache, blurred vision in the left eye, slurred speech, and transient weakness in the right upper limb. These symptoms began after a long-distance walk, around 8 km. His past medical, surgical, social, and family history were unremarkable, and with no stigmata of connective tissue disease and no history of altered level of consciousness. He had no history fevers, night sweating or use of illicit drug or any chronic prescribed medications.

On examination, the patient was alert, conscious, and in good general condition, with vital signs within normal limits. There were no palpable neck masses, carotid thrills, or bruits, and peripheral pulses were intact in both upper and lower extremities without radio-radial or radio-femoral delay. Neurological assessment revealed a positive Pulfrich phenomenon (a neuro-ophthalmic effect where 2-D objects appear three-dimensional due to small differences in signal transmission time between the eyes and the visual cortex) [8] in the left eye. Extraocular movements were intact, and motor examination showed normal power, tone, and reflexes, with no sensory deficits. Laboratory workup revealed normal hemoglobin, ESR, CRP, HbA1c, and lipid profile values. A CT angiography performed at the referring hospital revealed bilateral carotid artery dissection and development of a pseudoaneurysm in the cervical portion of the right internal carotid artery (ICA). The patient was started on a regimen of warfarin—5 mg for three days, followed by 4 mg for four days with a target INR of 2–3—before being referred to our institution.

**Fig. 1:**
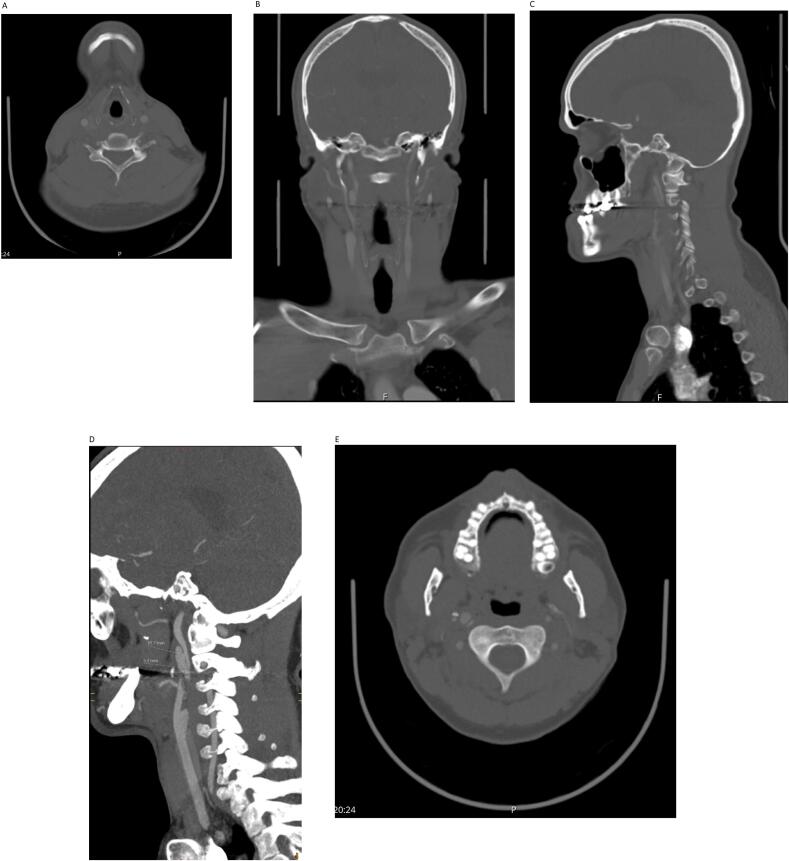
A: CT angiography, axial view showing no dissection in both common carotid arteries. B: CT angiography with coronal reconstruction showing right ICA pseudoaneurysm. C: CT angiography with sagittal reconstruction showing right ICA pseudoaneurysm. D: CT angiography with sagittal view showing right ICA pseudoaneurysm (15.2 × 5.3 mm). E: Axial cut showing dissection in the right ICA.

Two months later, the patient was seen in our clinic, symptom-free. A repeat CT angiography was performed, which revealed an improvement of the dissection bilaterally, a completely resolved dissection in the left ICA and confirmed the presence of a right ICA pseudoaneurysm (Fig. 1A, B, C, D, E), [June 2019]. Warfarin was switched to clopidogrel 75 mg orally once daily.

**Fig. 2:**
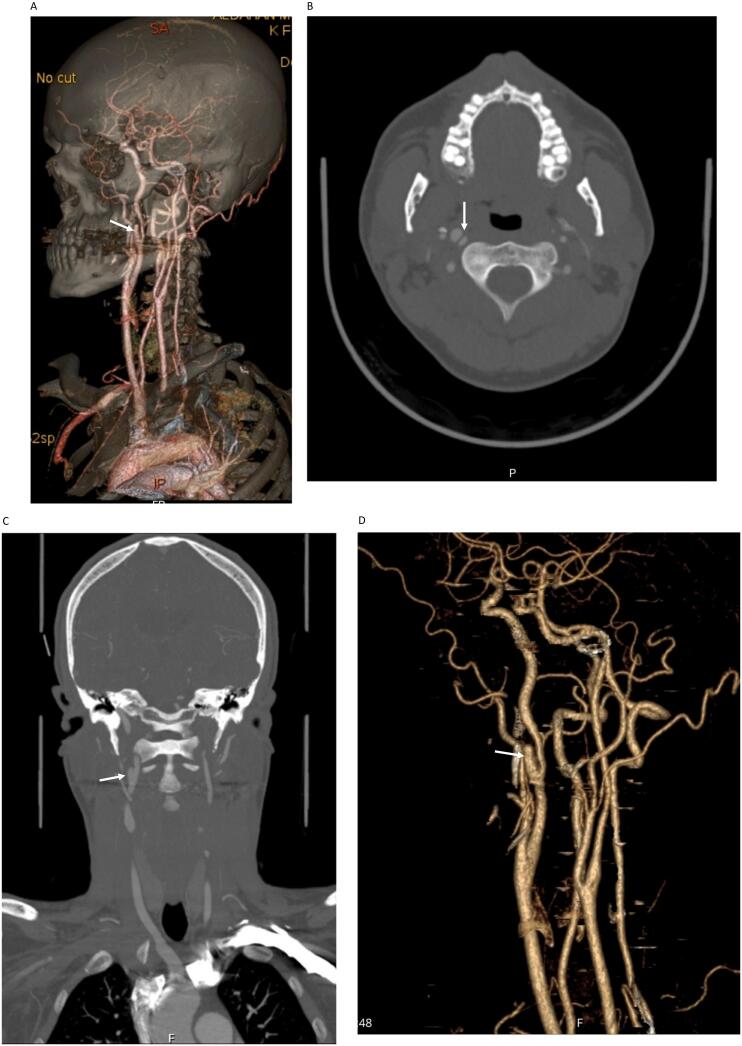
A: 3D volume rendering showing right ICA pseudoaneurysm (white arrow). B: CT angiography axial section showing no left ICA dissection, and evident right ICA dissection and pseudoaneurysm (white arrow). C: CT angiography coronal reconstruction showing right ICA pseudoaneurysm (white arrow). D: VR 3D reconstruction showing ICA pseudoaneurysm (arrow).

Upon follow-up four months later, the patient remained asymptomatic, and another CT angiography was performed, which showed complete resolution of left sided ICA dissection. There was also a slight reduction in the size of the right ICA pseudoaneurysm (Fig. 2A, B, C, D), [Oct 2019]. The case was discussed in our neurovascular multidisciplinary meeting, where it was agreed to continue the patient on conservative management with clopidogrel, and no intervention deemed necessary. The patient was given another appointment for follow-up with a new CT angiography in the following years.

**Fig. 3:**
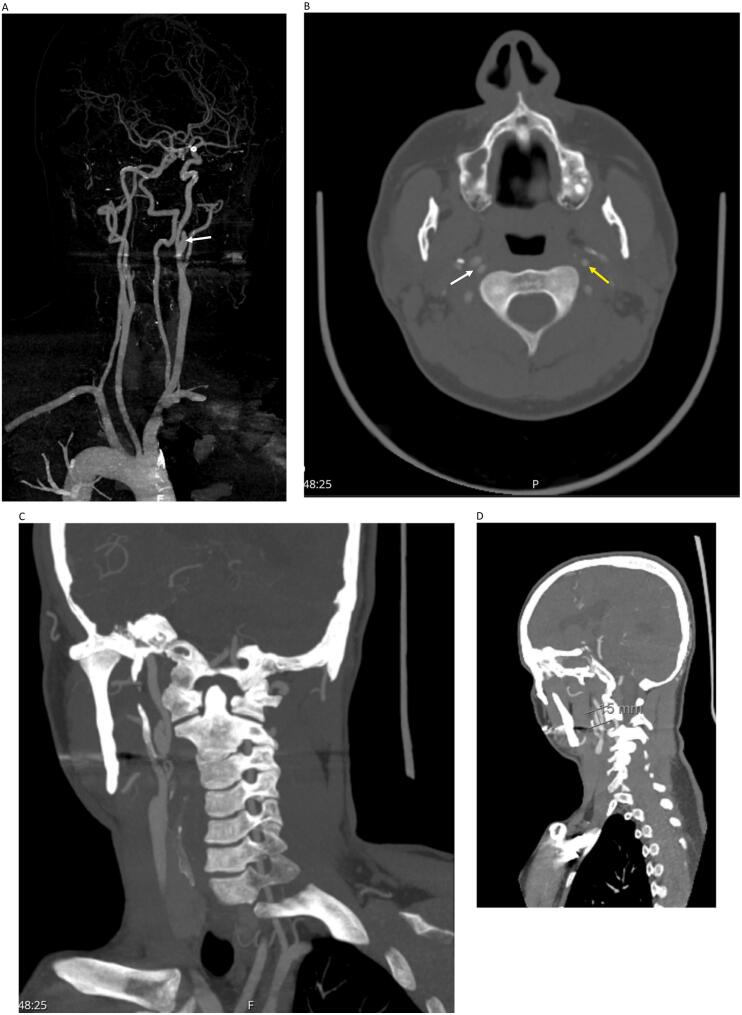
A: 3D reconstruction showing persistent right ICA pseudoaneurysm (arrow). B: CT angiography axial section showing normally appearing left ICA (yellow arrow) and dissected right ICA (white arrow). C: Maximum intensity projection showing right ICA pseudoaneurysm. D: Sagittal reconstruction with pseudoaneurysm size 15 mm.

**Fig. 4:**
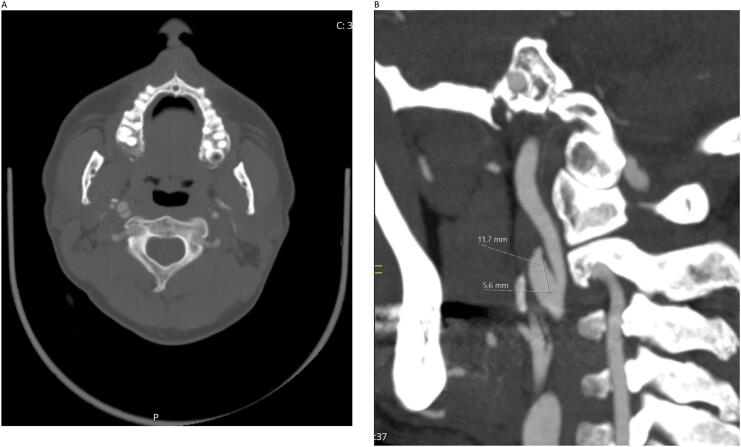
A: CT angiography coronal cut showing normally appearing left ICA with no dissection, with right ICA dissection. B: Axial cut. C: Smaller 11.7 mm right ICA pseudoaneurysm.

**Fig. 5:**
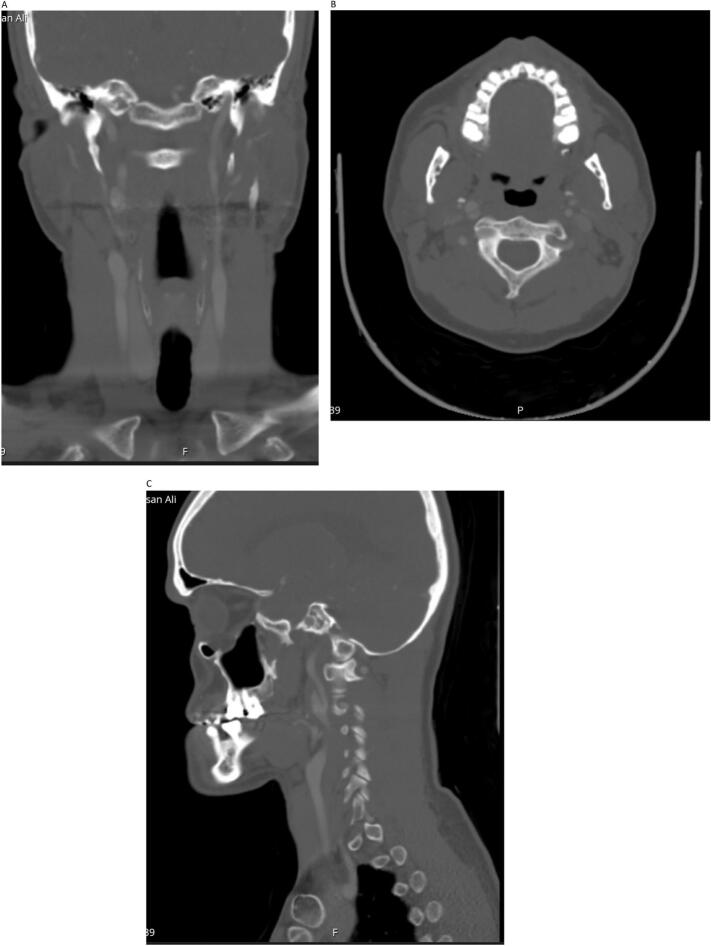
A: CTA coronal reconstruction showing shrinking right ICA pseudoaneurysm. B: CTA right ICA dissection. C: CTA sagittal view right ICA pseudoaneurysm with thrombosis.

Two CT angiography scans performed during two follow-ups over the following two years showed a patent left ICA with normal appearance. However, the right ICA pseudoaneurysm persisted but with stable size (Fig. 3A, B, C, D, E, F), [July 2021]. Two years later, a subsequent CT angiography revealed a completely normal left ICA and a reduction in the size of the right ICA pseudoaneurysm (Fig. 4A, B, C), [Oct 2023]. To date, five years later, the patient remained asymptomatic and continues to follow up regularly with the last CT angiography showing stable findings (Fig. 5A, B, C), [September 2024].

## 3 Discussion

Bilateral spontaneous carotid artery dissection, while uncommon, can occur in exceedingly rare circumstances [4]. The initial presentation of our patient with visual symptoms and contralateral arm weakness reflects mainly symptomatic left-sided dissection.

Conservative management with antiplatelet therapy alone proved to be an effective strategy, in line with the Society for Vascular Surgery guidelines [9]. The CADISS trial randomized 250 patients with extracranial carotid or vertebral dissection to antiplatelet or anticoagulant therapy and found no significant difference in stroke recurrence between groups at 3 months, and this was considered in the patient's medical management plan [10]. A meta-analysis by Sader et al. demonstrated a reduction in intracranial hemorrhage or death within the first 3 months when using antiplatelet therapy [11]. However, this finding was not statistically significant when the analysis was limited to only studies of high methodological quality [11]. Therefore, antiplatelet therapy is arguably considered the safer option, particularly in cases with intracranial extension or large infarcts where the bleeding risk may be higher.

The natural history of carotid artery dissection is generally benign, with the majority of patients having good functional outcomes [12]. In our patient, follow-up imaging revealed spontaneous resolution of the left ICA dissection and a pseudoaneurysm formation in the right ICA, which improved in later scans. Hence, this case comes in support of conservative therapy in patients presenting with seemingly mild or transient neurological symptoms, especially in young patients lacking major anatomical constraints such as stenosis. Similarly, Townend et al. reported one case of a 51-year-old patient with bilateral carotid artery dissection that was treated successfully using clopidogrel monotherapy [4]. Other reports have also found positive outcomes with conservative management [7].

The underlying mechanisms behind the formation of carotid pseudoaneurysms remain incompletely understood. Surgical and endovascular interventions are traditionally the primary modalities for managing carotid pseudoaneurysms [13]. Nonetheless, multiple studies have documented successful outcomes with conservative management. Xue et al. and Zhou et al. reported satisfactory results using oral anticoagulants and antiplatelets, with a similarly notable reduction in pseudoaneurysm size [14,15]. However, conservative management alone may carry risks, as other studies have cautioned against its use due to the possibility of delayed rupture [16].

## 4 Conclusion

Bilateral spontaneous carotid artery dissection is a rare finding, and requires a high index of suspicion, and prompt early neurological assessment and radiological investigation. Conservative management with antiplatelets is associated with positive outcomes in carefully selected patients. Larger controlled studies are needed to clarify optimal management strategies, particularly for carotid artery dissection with pseudoaneurysm formation.

## Author contribution

Ahmed Almumtin, Principal investigator, Idea, designs, writing and review.

Feddah Almutairi: Literature review, and scientific data.

Amro Hajja: Literature review and data collection.

Nancy Darwish: literature review and referencing.

Samer Koussayer: Overall supervision.

## Consent

Written informed consent was obtained from the patient for publication of this case report and accompanying images. A copy of the written consent is available for review by the Editor-in-Chief of this journal on request.

## Ethical approval

King Faisal Specialist Hospital and Research Centre waived the need for IRB approval due to the absence of patient identification in the study's enrolled participant.

## Guarantor

Ahmed Almumtin.

## Funding

Not applicable.

## Conflict of interest statement

The authors have no competing interests.
